# Incidence and risk factors for decreased range of motion of the knee joint after surgery for closed tibial plateau fracture in adults

**DOI:** 10.1186/s13018-021-02700-2

**Published:** 2021-09-07

**Authors:** Junyong Li, Junzhe Zhang, Kuo Zhao, Yanbin Zhu, Hongyu Meng, Zhucheng Jin, Dandan Ye, Wei Chen, Yingze Zhang

**Affiliations:** 1 Department of Orthopaedic surgery, The 3rd Hospital of Hebei Medical University, Shijiazhuang, 050051 Hebei P. R. China; 2 Key Laboratory of Biomechanics of Hebei Province, Shijiazhuang, 050051 Hebei P. R. China; 3 Orthopaedic Institution of Hebei Province, Shijiazhuang, 050051 Hebei P. R. China; 4 NHC Key Laboratory of Intelligent Orthopaedic Equipment, Shijiazhuang, 050051 Hebei P. R. China; 5 Hebei Orthopedic Clinical Research Center, Shijiazhuang, 050051 Hebei P. R. China; 6 The First Hospital of Shijiazhuang City, Shijiazhuang, 050000 Hebei P. R. China

**Keywords:** Tibial plateau fractures, Decreased, Range of motion, Incidence, Risk factor

## Abstract

**Purpose:**

The aim of this study was to quantify the incidence of and identify independent risk factors for decreased range of motion (ROM) of the knee joint after surgery for closed tibial plateau fractures in adults.

**Methods:**

This retrospective study was performed at the trauma centre in our hospital from January 2018 to December 2019. Data from adult patients with tibial plateau fractures treated by surgery were extracted from the electronic medical records. A total of 220 tibial plateau fracture patients were enrolled. We extracted the patients’ demographic characteristics, fracture characteristics, and surgery-related variables. Univariate and multivariate logistic regression models were used to investigate the potential independent risk factors.

**Results:**

Fifty-seven patients developed decreased ROM of the knee joint at the 1-year follow-up in this study. The overall incidence was 25.9%. The independent predictors of decreased ROM after surgery, as identified in the multivariate analysis, were orthopedic polytrauma (odds ratio = 3.23; 95% CI = 1.68–6.20; *p* = 0.000), fracture type (Schatzker V-VI) (odds ratio = 2.52; 95% CI = 1.16–5.47; *p* = 0.019), and an open reduction and internal fixation approach (odds ratio = 2.10; 95% CI = 1.07–4.12; *p* = 0.031).

**Conclusions:**

The study confirmed that patients with orthopaedic polytrauma, more complex fractures and those treated with open reduction and internal fixation (ORIF) surgery were more likely to suffer decreased ROM of the knee joint 1 year after surgery.

## Background

Surgical treatment is the most common method to treat tibial plateau fractures. With the continuous development of surgical techniques and internal fixation materials, an increasing number of studies have reported good outcomes after surgical treatment of tibial plateau fractures. However, there are still postoperative complications that affect the short- or long-term prognosis of patients experiencing tibial plateau fracture [1, 2]. Among these complications, decreased range of motion (ROM) of the knee joint is one of the important factors affecting function after surgery. Lack of the ROM of the knee joint will affect the function of the joint, which will have an important impact on the work and life of the patient after surgery, such as reduced physical flexibility and reduced motor function [3]. Previous studies reported that the incidence of joint stiffness (ROM < 90°) was up to 7% after tibial plateau fractures, so the incidence of a decreased ROM (less than normal) of the knee joint was higher after surgery [4, 5]. Another study reported that 21% of patients still had residual flexion deformity after 1 year of tibial plateau fracture open reduction and internal fixation (ORIF) surgery, which severely affected their return to work, especially manual workers or athletes. Some patients even needed a second surgical intervention to improve knee joint function. Although there have been many studies on ROM after knee joint surgery in the past, most of them have focused on the decreased ROM after total knee replacement and ligament injury [6–11]. To our knowledge, there have been no studies specifically targeting decreased ROM of the knee joint after surgery for tibial plateau fractures.

Given the above information, we designed this retrospective study with two aims: first, to describe the incidence of decreased ROM of the knee joint after surgery at the 1-year follow-up and second, to investigate the related risk factors for the occurrence of decreased ROM of the knee joint.

## Methods

Our retrospective investigation identified adult patients (18 years or older) with acute closed tibial plateau fractures treated by surgery at the trauma center in our hospital from January 2018 to December 2019 who had a postoperative follow-up of at least 1 year. The exclusion criteria were open fractures, pathological fractures caused by other diseases, treatment with conservative methods, patients with a previous history of knee osteoarthritis, incomplete medical data, incomplete follow-up, fractures around a prosthesis, and postoperative clinical adverse events such as infection, nonunion, or delayed union.

### Perioperative management

We performed temporary plaster external fixation and highly immobilized the affected limb before surgery. Prophylactic antibiotics were administered intravenously 30 min before surgery according to guideline recommendations [12]. All patients were provided a list of rehabilitation exercises after surgery, which included a continued emphasis on the ROM of the knee joint and muscle-strengthening exercises; furthermore, weight bearing was kept to a minimum with the use of two crutches for 8 to 12 weeks in all patients. During their stay in the hospital, patients are guided by a specialized rehabilitation trainer for functional exercise. After discharge, researchers provided continued functional exercise guidance to patients through telephone follow-ups.

### Postoperative follow-up

All patients returned to the hospital at 3 months, 6 months, and 1 year after surgery. X-ray examination was performed to confirm fracture healing, and postoperative knee joint recovery was recorded by the researchers. Clinical function and radiographic outcomes were evaluated, and the ROM of the knee joint was recorded by two specially trained orthopaedic surgeons. The specific method of measurement was to use a universal goniometer to measure the ROM. By aligning the fixed arm and movable arm of the goniometer with specific bone marks on both sides of the joint, the degree of ROM could be measured in degrees [13]. To accurately analyze the factors associated with a decreased ROM of the knee joint, we used data from 12 months postoperation for statistical analysis. Patients with complete data were included in this retrospective investigation.

### Definition of decreased ROM of the knee joint

We performed knee hospital for special surgery (HSS) [14] and American Knee Society knee score (KSS) [15] assessments on each patient who returned to the hospital for review, and a dedicated team of surgeons was assigned to measure the ROM of the knee joint. The normal ROM of the knee joint is approximately 0–150°, as previously reported in the literature [16]. According to the HSS and KSS, the normal ROM of the knee joint after surgery should be 0–144° and 0–125°, respectively. Referring to the minimum value of the normal ROM, we defined a decreased ROM as an ROM of the knee joint that was less than 125° at the 1-year follow-up.

This study was approved by the Institutional Review Board of our hospital before its commencement.

### Data collection and variables

All information of interest was extracted from the electronic medical records, including the demographic characteristics, injury-related variables, and surgery-related variables. Demographic information, including age, sex, weight, height, chronic diseases (hypertension, diabetes mellitus, cerebrovascular disease, chronic heart disease), residential area (rural or urban), history of any surgery, allergic history, smoking status, and alcohol consumption, was extracted and documented.

Body mass index (BMI) was calculated by dividing weight (kg) by the square of height (meters), and BMI was divided into four groups according to Chinese standards [17]: normal, 18.5–23.9; underweight, < 18.5; overweight, 24–27.9; obese and morbidly obese, ≥ 28.

The fracture characteristic variables included the injury mechanism (low or high energy), injury type (close or open), side involved, fracture classification (Schatzker classification system), and orthopedic polytrauma (more than one site of fracture). Low-energy injuries were defined as falls from standing height, while high-energy injuries were defined as traffic accidents, falls from a height and sports injuries.

Surgery-related variables included preoperative duration, anesthesia pattern, ASA grade (American Society of Anaesthesiologists), presence of deep vein thrombosis (intermuscular vein thrombosis), operative duration, closed reduction, and minimally invasive internal fixation or open reduction internal fixation (ORIF), intraoperative blood loss, intraoperative blood transfusion, and bone grafting. The preoperative duration was defined as the time from injury to surgery and divided into two groups: 1, ≤ 7 days and 2, > 7 days. Anesthesia patterns were divided into regional anesthesia and general anesthesia. The operative duration was also divided into two groups: 1, ≤ 120 min and 2, > 120 min. Intraoperative blood loss was divided into two groups: 1, ≤ 400 ml and 2, > 400 ml. The bone grafting pattern was divided into autografts and allografts.

### Minimally invasive surgical technique

**Fig. 1:**
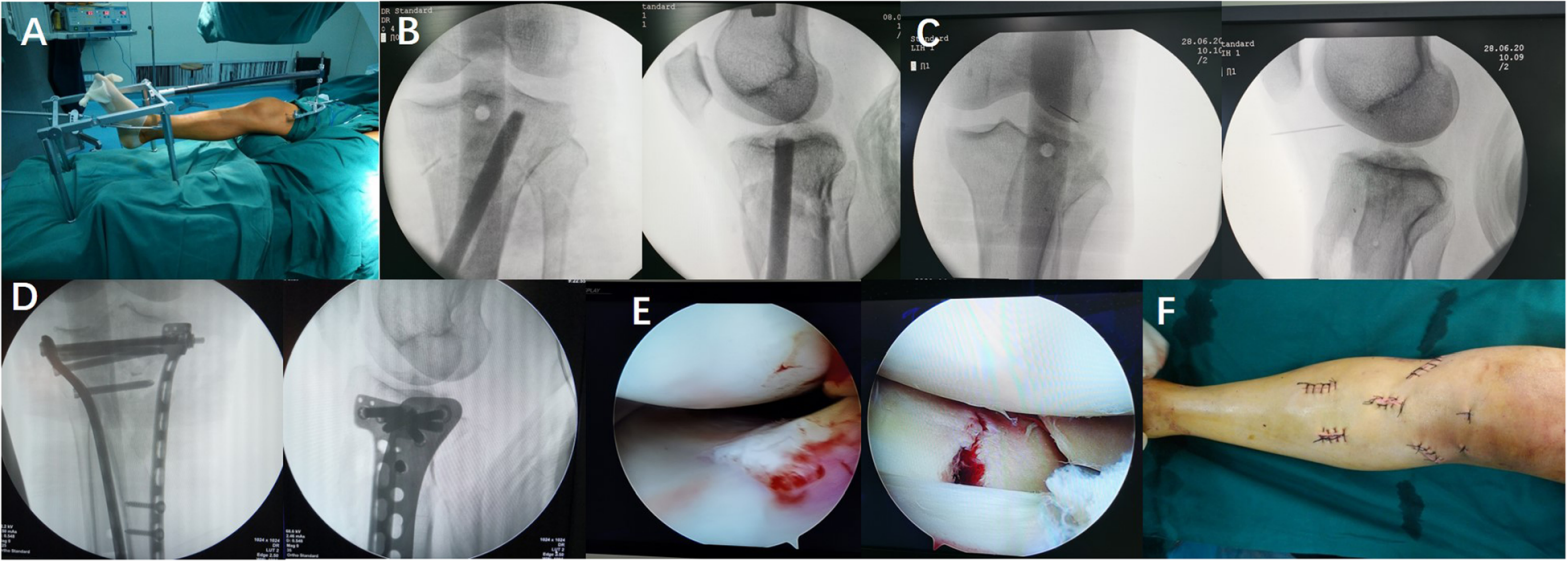
Close reduction minimally invasive surgery procedures. **A** Demonstration of the interaction forces acting on the distal tibia and supracondylar femur. **B** Percutaneous reduction of depressed fragments with the top rod. **C** The insertion of autogenous bone graft to support the articular surface. **D** The position of fracture reduction and internal fixation was confirmed to be satisfactory. **E** After fluoroscopic reduction and internal fixation, arthroscopy showed that the medial and lateral articular surfaces were almost anatomically reduced. **F** Seven minimal incisions after the operation, four for percutaneous insertion of plates, one for indirect reduction of depressed fragments, and two for arthroscopic inspection

It should be noted that in the closed reduction and minimally invasive internal fixation group, we used a double reverse traction reduction device and special tools that were originally designed at our centre. The surgical procedures have been described in detail in previous literature [18]. For the treatment of articular surface compression displacement, we determined the collapse site according to preoperative X-ray and CT scans. The depressed fragments were elevated with a customized bone tamp via the tunnel created by step drills. The reduction process was monitored by anteroposterior and lateral positions of C-arm fluoroscopy, and the bone compression block was tapped. After the collapse and displacement of the articular surface was reduced, autologous iliac bone was taken and inserted into the bone tunnel to support the subchondral bone and articular surface. For widening the displacement of the articular surface, 1–2 compression bolts with special slots were inserted into the nearest proximal holes of the two plates to restore the width of the tibial plateau. Finally, minimally invasive percutaneous plate osteosynthesis (MIPPO) was performed using a locking compression plate (LCP) designed for the proximal tibia in both anteroposterior and lateral fluoroscopy (Fig. 1). The bidirectional reductor was removed and arthroscopic evaluations were carried out to check the quality of intra-articular reduction. Satisfactory reduction of the fracture was observed arthroscopically. (Fig. 1E)

### Statistical analysis

According to our pre-statistical method [19], Student’s *t* test and the Mann-Whitney *U* test were used for continuous variables (depending on whether the data for the variable were normally distributed), and the threshold for significance was *p* < 0.05. A univariate analysis was used to evaluate the relationship between each categorical variable and the decreased ROM of the knee joint. Then, the variables that were significant in the univariate analyses to predict the decreased ROM of the knee joint were included in the multivariate logistic regression analysis model, and the independent predictors of the decreased ROM of the knee joint were finally determined. The goodness of fit of the model was tested using the Hosmer-Lemeshow test, and *p* > 0.05 was an acceptable goodness of fit.

## Results

### Characteristics of the study sample

**Fig. 2:**
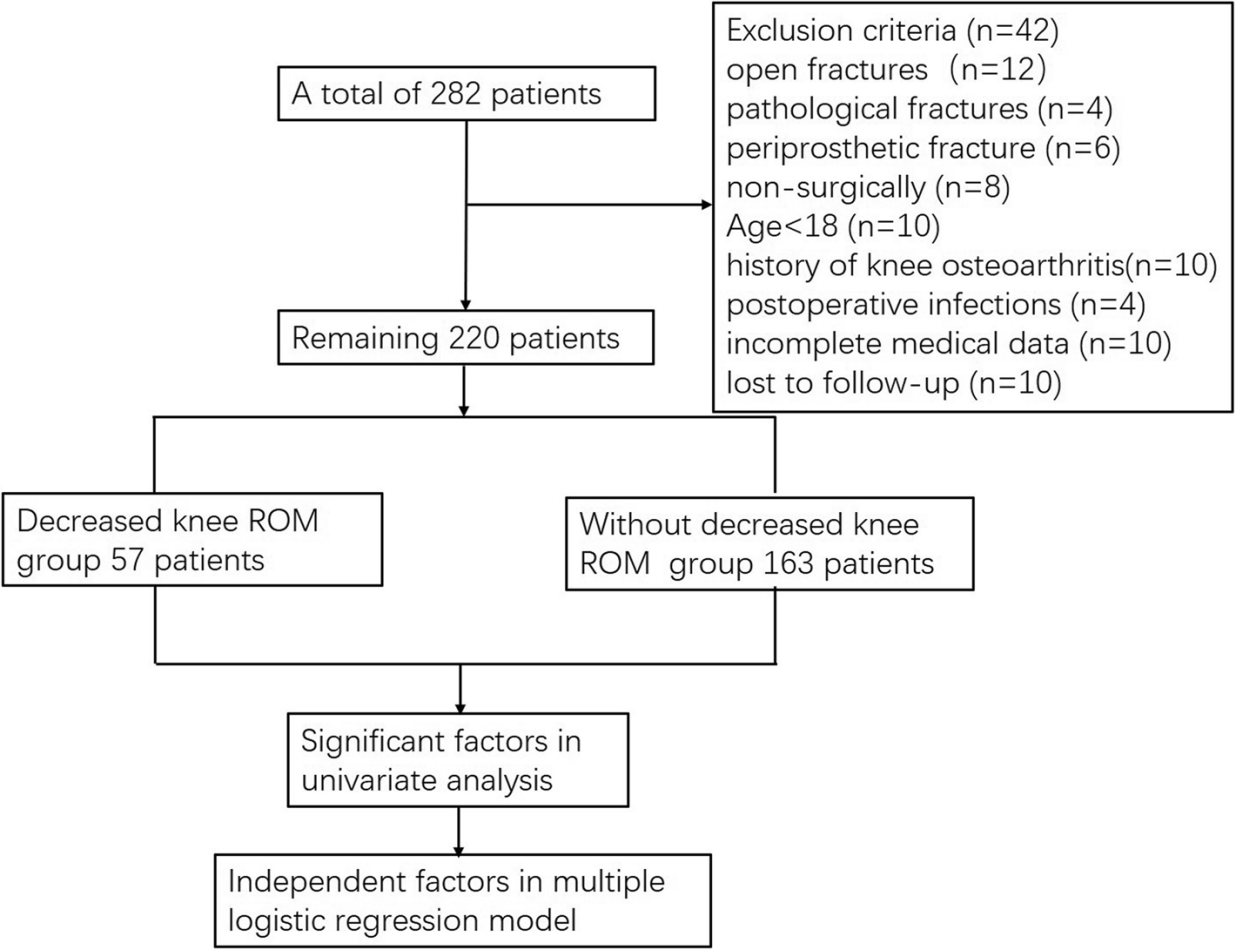
The flow chart for the selection of study participants

Overall, the data of 282 patients with tibial plateau fractures were collected. Ten patients were excluded because they were under 18 years old; 10 had incomplete medical data; 10 were lost to follow-up; 12 had open fractures; 4 had pathological fractures; 6 cases were diagnosed as periprosthetic fractures; 8 underwent nonsurgical treated; 10 had a previous history of knee osteoarthritis; and postoperative infection occurred in 4 patients. However, no fractures progressed to nonunion. Finally, data from 220 patients were analyzed in this study (Fig. 2). Of these patients, 154 were male and 66 were female, with a mean age of 42.2 years (range from 18 to 74). Left-side tibial plateau fractures were involved in 125 patients, and right-side fractures were involved in 95 patients. The injuries of 136 patients were caused by a high-energy damage mechanism. There were 85 patients injured in traffic accidents, 23 patients who fell from a height, 13 patients with sporting injuries and 11 patients who had other injuries. Of the 220 patients, 80 had more than one fracture (which we defined as orthopaedic polytrauma). According to the Schatzker classification system, the corresponding numbers of type I, II, III, IV, V, and VI fractures were 57, 74, 24, 27, 16, and 22, respectively. Ninety-seven patients were treated with close reduction and minimally invasive internal fixation, and 123 patients were treated with ORIF.

### Characteristics of decreased ROM of the knee joint

According to the assessment form, the average ROM of the knee joint score of the patients reviewed at least 1 year after surgery was 136°. The ROM score ranged from 64 to 144°. After a minimum of 1 year follow-up, there were 57 patients with varying degrees of decreased ROM, accounting for 25.9% of the total participants.

There were no significant differences between the patients with decreased ROM of the knee joint and the patients without decreased ROM of the knee joint in terms of age (41.2 vs 42.6 years, *p* = 0.067), intraoperative blood loss (303.5 vs 263.5 ml, *P* = 0.817) or operation duration (141.7 vs 145.4 min, *P* = 0.106). However, a significant difference in the length of the preoperative stay was observed (7.7 vs 6.3 days, *P* = 0.023). Patients with decreased ROM of the knee joint had a prolonged mean length of hospitalization (7.7 days) compared to those without decreased ROM of the knee joint (24.2 vs 16.5 days, *P* < 0.001). These results are presented in Table 1.

**Table 1:** Comparison of continuous variables in patients with and without decreased ROM

Variables	Patient without limited ROM (mean, standard deviation) (***n*** = 163)	Patient with limited ROM (mean, standard deviation) (***n*** = 57)	***P***
Age (years)	42.6 (9.8)	41.2 (11.3)	0.400
Preoperative stay (days)	6.3 (4.3)	7.7 (6.3)	0.057
Intraoperative blood loss (ml)	287.8 (184.7)	303.5 (262.6)	0.623
Operation duration (minutes)	145.4 (59.9)	141.7 (45.8)	0.667
Hospital stay (days)	16.5 (7.8)	24.2 (29.7)	0.003 *

*Significant variables

### Univariate and multivariate analyses

In univariate analysis, 25 variables including age, comorbidity, allergy history, place of residence, smoking, drinking, body mass index, injury mechanism, fracture type, thrombosis of lower limb, interval, anesthesia methods, ASA, operation method, whether intraoperative bone graft, operation time, intraoperative bleeding, and blood transfusion are included in the model. Factors that significantly increased the risk of decreased ROM of the knee joint in univariate analysis were preoperative duration, deep vein thrombosis, orthopedic polytrauma, fracture type (Schatzker V-VI), and ORIF (Table 2).

**Table 2:** Univariate analyses of risk factors associated with decreased ROM after surgery of tibial plateau fracture

Variables	Number (%) of limited ROM (*n* = 57)	Number (%) of without limited ROM (*n* = 163)	***P***
**Age (years)**			0.599
**18–40**	25 (43.9)	65 (39.9)	
**41–60**	32 (56.1)	98 (60.1)	
**Diabetes mellitus**	5 (8.8)	17 (10.4)	0.720
**Hypertension**	11 (19.3)	27 (16.6)	0.638
**Cerebrovascular disease**	0 (0.0)	3 (1.8)	0.302
**Chronic heart disease**	3 (5.3)	7 (4.3)	0.762
**History of any surgery**	7 (12.3)	17 (10.4)	0.700
**Allergy to any medications**	1 (1.8)	11 (6.7)	0.153
**Living area**			0.555
**Rural**	33 (57.9)	87 (53.4)	
**Urban**	24 (42.1)	76 (46.6)	
**Current smoking**	7 (12.3)	36 (22.1)	0.108
**Alcohol consumption**	4 (7.0)	27 (16.6)	0.075
**Preoperative duration (days)**			0.030*
**1–7**	32 (56.1)	117 (71.8)	
**> 7**	25 (43.9)	46 (28.2)	
**Deep vein thrombosis**	14 (24.6)	21 (12.9)	0.038*
**Intermuscular vein thrombosis**	1 (1.8)	2 (1.2)	0.768
**BMI (kg/m**^**2**^**)**			0.331
**18.5–23.9**	19 (33.3)	62 (38.0)	
**< 18.5**	1 (1.8)	0 (0.0)	
**24–27.9**	24 (42.1)	70 (42.9)	
**≥ 28.0**	13 (22.8)	31 (19.0)	
**Anesthesia (general)**	35 (61.4)	97 (59.5)	0.802
**Orthopedic polytrauma**	37 (64.9)	55 (33.7)	0.000*
**Mechanism (high-energy)**	40 (70.2)	96 (58.9)	0.131
**Fracture type (Schartzker)**			0.004*
**I–IV**	40 (70.2)	142 (87.1)	
**V–VI**	17 (29.8)	21 (12.9)	
**Closed or open reduction procedures**			0.027*
**ORIF**	39 (68.4)	84 (51.5)	
**CRMIIF**	18 (31.6)	79 (48.5)	
**Bone grafting (yes)**	16 (28.1)	44 (27.0)	0.875
**Bone graft type**			0.943
**Autograft**	11 (19.3)	32 (19.6)	
**Allograft**	5 (8.8)	12 (7.4)	
**Operative duration (min)**			0.726
**1–120**	24 (42.1)	73 (44.8)	
**> 120**	33 (57.9)	90 (55.2)	
**Intraoperative blood loss (ml)**			0.770
**1–400**	51 (89.5)	148 (90.8)	
**> 400**	6 (10.5)	15 (9.2)	
**ASA class**			0.696
I	10 (17.5)	23 (14.1)	
II	35 (61.4)	110 (67.5)	
III or above	12 (21.1)	30 (18.4)	
**Intraoperative blood transfusion**	2 (3.5)	5 (3.1)	0.870

*Abbreviations*: *ORIF* open reduction and internal fixation, *CRMIIF* closed reduction and minimally invasive internal fixation, *ASA* American Society of Anesthesiologists, *BMI* body mass index,

*Significant variables

The multivariate analysis results showed that orthopedic polytrauma (OR = 3.231; 95% CI 1.684–6.196; *p* = 0.000), fracture type (Schartzker V–VI) (OR = 2.521; 95% CI 1.162–5.471; *p* = 0.019), and ORIF (OR = 2.09; 95% CI 1.068–4.124; *p* = 0.031) were associated with decreased ROM of the knee joint (Table 3). The Hosmer-Lemeshow test showed adequate fitness (*χ*2 = 8.168; *p* = 0.086), and the Omnibus tests of model coefficients also showed the model made sense overall (*χ*2 = 26.315; *p* = 0.000).

**Table 3:** Multivariate analysis of factors associated with decreased ROM after surgery of tibial plateau fracture

Variables	OR	95%CI (lower limit)	95%CI (upper limit)	***P***
**Orthopaedic polytrauma**	3.231	1.684	6.196	0.000*
**ORIF**	2.099	1.068	4.124	0.031*
**Fracture type (Schartzker V–VI)**	2.521	1.162	5.471	0.019*
**Preoperative duration (days)**	1.692	0.860	3.330	0.128
**Deep vein thrombosis**	1.498	0.649	3.460	0.344

*Abbreviations*: *ORIF* open reduction and internal fixation

*Significant variables

## Discussion

To determine the incidence and risk factors for decreased ROM of the knee joint after surgery for closed tibial plateau fractures, we conducted this study and showed that the overall incidence of decreased ROM of the knee joint was 25.9% after a minimum 1 year follow-up in adults. Independent risk factors included orthopedic polytrauma, fracture type (Schatzker V–VI), and ORIF.

Orthopaedic polytrauma as a significant risk factor for decreased ROM of the knee in fractures of the tibial plateau has been confirmed in the previous literature [20, 21]. Such patients have a 3–6 times greater risk of experiencing decreased ROM after surgery than patients with a single fracture. Their results were similar to ours (*P* = 0.000 or *P* = 3.231). At the follow-up conducted approximately 1 year after surgery, the percentage of decreased ROM of the knee joint in patients with orthopedic polytrauma was 40.4% (37/92), which was much higher than that in patients with a single site of tibial plateau fracture (15.6%; 20/128).

Thus, orthopedic polytrauma is a factor that affects the ROM of the knee joint after surgery due to the following. First, to allow patients with orthopedic polytrauma to safely tolerate surgical anesthesia, compound injuries in other parts might be treated as a priority before surgery at the main fracture site. Thus, the time interval before surgery and bed occupancy time increased [22, 23]. Second, if patients needed surgical treatment for multiple fracture sites and could not undergo multiple surgical procedures at the same time, patients were treated with staged surgical treatment. This also prolonged the patient’s hospital stay and bed occupancy time and delayed the patient’s rehabilitation exercise. Finally, due to the high incidence of complications, such as thrombosis and infection, patients with orthopaedic polytrauma were treated with limb immobilization, and the postoperative treatment period was longer. Thus, the postoperative care and rehabilitation plan was more complex and full of uncertainty in this circumstance [24]. Considering the adverse effect of the above factors, we should pay more attention to patients with orthopedic polytrauma and complete comprehensive evaluations before surgery, thereby selecting the best surgery time, formulating a reasonable postoperative rehabilitation plan, and minimizing the adverse impact of orthopaedic polytrauma on the ROM of the knee joint after surgery.

The concept of fracture type (Schatzker V–VI) affecting the ROM of the knee joint after surgery has also been well studied in previous investigations. Yao et al. [25] discovered that the more complex the fracture type was, the worse the clinical outcome. Hap et al. reported that patients with Type I–IV injuries had a significantly higher SF-36 score than patients with Type V-VI injuries [26]. Bradley et al. [21] confirmed that patients with Schatzker V–VI fractures were more than 3 times more likely to require surgical intervention because of knee stiffness after surgery.

In our study, patients with Schatzker type V–VI fractures were more than twice as likely to have a decreased ROM compared with patients with other fracture types (OR, 2.521; 95% CI, 1.16–5.47; *P* = 0.019), and this estimate was comparable to those reported in previous studies. Schatzker V–VI tibial plateau fractures are complex injuries that are difficult to treat. This kind of fracture is characterized by the involvement of internal and external articular surfaces accompanied by metaphyseal separation of joint fragments. In addition, these fractures are usually caused by high-energy trauma [27, 28]; thus, damage to the surrounding soft tissue is severe and the incidence of postoperative joint stiffness increases [29, 30]. It is also important to point out that this type of fracture is often associated with a number of accessory structure injuries, such as ligamentous and meniscal tears, in addition to cartilaginous damage. Stahl et al. [31] confirmed that 30% of these fractures were associated with a torn meniscus that required surgical management. Mui et al. [32] found that meniscal tears were seen in 22% of these fractures, and ligament disruption was seen in 43% to 80% of these fractures. These fracture-related complications increased the incidence of decreased ROM of the knee joint after surgery. This conclusion was also verified in our study. Among the 38 patients with Schatzker V–VI fractures in our survey, 17 patients developed decreased ROM postoperatively, indicating that the incidence was 44.7% (17/38). In summary, the postoperative recovery of joint function in patients with Schatzker V–VI fractures requires increased attention from medical staff. To avoid the postoperative decreased ROM of the knee joint, early professional guidance for functional limb exercise is necessary.

In our study, compared with conventional closed reduction and minimally invasive internal fixation, ORIF was found to be one of the independent risk factors (OR = 2.099; 95% CI, 1.068–4.124; *P* = 0.031) for decreased ROM of the knee joint after surgery. We designed percutaneous surgery procedures, including the use of an originally designed double reverse traction reduction device and special tools, which could promote the close reduction of tibial plateau fractures. Compared with ORIF, our minimally invasive surgery had the following advantages. First, this surgical technique required only a few 2–3 cm surgical incisions, so there were fewer soft tissue complications, such as postoperative surgical site infection and skin necrosis. In our study, the wound infection rate following close reduction minimally invasive surgery was 1.02% (1/98) and that of ORIF was 2.4% (3/126). Second, the double reverse traction reduction device could be used for continuous traction; therefore, the partial reduction of the fracture could be achieved by traction of the joint capsule and the compression of soft tissue without the need to open the joint capsule. Regarding the collapse and widening displacement of the articular surface, we could achieve satisfactory reduction through the use of special surgical instruments, such as a top rod and compression bolts, and the results could be verified under postoperative arthroscopy. Furthermore, because of the use of bone grafts under the articular surface and compression bolts, fracture reduction was very stable, and patients did not need limb immobilization after surgery, thereby enabling patients to carry out early functional exercise and promote the recovery of the knee joint. Third, compared with ORIF, our surgical reduction was faster, the operation time was shorter, and the blood loss was less, which was also reported in previous studies [18, 33–35]. In practice, our minimally invasive surgical techniques and reduction tools are suitable for most types of tibial plateau fractures. This original surgical technique provides a new approach to the clinical minimally invasive treatment of tibial plateau fractures with good clinical results.

This study had several limitations. First, the main limitation of the retrospective cohort studies is that the research question was formulated after data collection therefore, potential confounders and variables may have been missed from the model. Second, the follow-up time of this study was relatively short, with an average of 12 months. In the future, prospective outcome studies should be conducted to further explore the mid-term or long-term effect of these risk factors on the postoperative ROM of the knee joint.

## Conclusion

The study confirmed that patients with orthopedic polytrauma, more complex fractures and those treated with open reduction and internal fixation (ORIF) surgery, were more likely to suffer a decreased ROM of the knee joint 1 year after surgery.

## Data Availability

The data and materials contributing to this article may be made available upon request by sending an e-mail to the first author.

## Abbreviations

ROM: Range of motion
ORIF: Open reduction and internal fixation
ASA: American Society of Anaesthesiologists
BMI: Body mass index
